# Near-infrared spectroscopy and machine learning algorithms for rapid and non-invasive detection of Trichuris

**DOI:** 10.1371/journal.pntd.0011695

**Published:** 2023-11-13

**Authors:** Tharanga N. Kariyawasam, Silvia Ciocchetta, Paul Visendi, Ricardo J. Soares Magalhães, Maxine E. Smith, Paul R. Giacomin, Maggy T. Sikulu-Lord

**Affiliations:** 1 School of the Environment, Faculty of Science, The University of Queensland, Brisbane, Queensland, Australia; 2 School of Veterinary Science, Faculty of Science, The University of Queensland, Gatton, Queensland, Australia; 3 Institute for Molecular Bioscience, The University of Queensland, Brisbane, Queensland, Australia; 4 Children’s Health and Environment Program, UQ Children’s Health Research Centre, Faculty of Medicine, The University of Queensland, Brisbane, Queensland, Australia; 5 Australian Institute of Tropical Health & Medicine, James Cook University, Cairns, Queensland, Australia; University of Liverpool, UNITED KINGDOM

## Abstract

**Background:**

*Trichuris trichiura* (whipworm) is one of the most prevalent soil transmitted helminths (STH) affecting 604–795 million people worldwide. Diagnostic tools that are affordable and rapid are required for detecting STH. Here, we assessed the performance of the near-infrared spectroscopy (NIRS) technique coupled with machine learning algorithms to detect *Trichuris muris* in faecal, blood, serum samples and non-invasively through the skin of mice.

**Methodology:**

We orally infected 10 mice with 30 *T*. *muris* eggs (low dose group), 10 mice with 200 eggs (high dose group) and 10 mice were used as the control group. Using the NIRS technique, we scanned faecal, serum, whole blood samples and mice non-invasively through their skin over a period of 6 weeks post infection. Using artificial neural networks (ANN) and spectra of faecal, serum, blood and non-invasive scans from one experiment, we developed 4 algorithms to differentiate infected from uninfected mice. These models were validated on mice from a second independent experiment.

**Principal findings:**

NIRS and ANN differentiated mice into the three groups as early as 2 weeks post infection regardless of the sample used. These results correlated with those from concomitant serological and parasitological investigations.

**Significance:**

To our knowledge, this is the first study to demonstrate the potential of NIRS as a diagnostic tool for human STH infections. The technique could be further developed for large scale surveillance of soil transmitted helminths in human populations.

## Author summary

The existing diagnostic tools for STH infections can be time consuming, expensive, less sensitive and some require trained personnel. These techniques are therefore not feasible for large scale programmatic surveillance. Novel surveillance tools that can be easily scaled up to guide mass drug administration (MDA) for communities at risk remain a priority. This study investigated the role of NIRS as a potential large scale surveillance tool for soil transmitted helminths using *Trichiuris muris* experimental mouse model. The technique is environmentally friendly because it neither requires reagents nor sample processing procedures to operate and it only takes 5–10 seconds to analyse a sample allowing thousands of samples to be assessed in a day by unskilled personnel. Our results demonstrate for the first time that infrared light coupled with machine learning has the potential to be developed into a large-scale surveillance tool for STH infections to assess impact of interventions and guide future elimination efforts.

## Introduction

More than 1.5 billion people around the world are at risk of contracting soil-transmitted helminths (STH). In areas with poor sanitation, STH are transmitted when contact occurs with soil contaminated with eggs from faeces of definitive hosts or food with eggs. Medically important species are whipworms (*Trichuris trichiura*), hookworms (*Necator americanus* and *Ancylostoma duodenale*) and roundworms (*Ascaris lumbricoides*) [1]. With over 267million pre-school and over 568 million school-age children infected with STH, the two groups remain the most vulnerable to STH infections [1]. *T. trichiura* alone infects an estimated 604–795 million people worldwide with a majority of them being pre-school and school aged children [2]. These infections are often linked to malnutrition, stunted growth, intellectual retardation, school absenteeism among infected children, [3] maternal anaemia for pregnant women as well as low birth weight and reduced survival for unborn babies [4]. Moreover, where prevalent is highest, the burden of STH negatively affects the economy [4,5].

Following the ingestion of eggs via contaminated food or hands, the eggs of *T*. *trichiura* hatch in the small intestines and adult worms live in the ascending colon and cecum up to one year [2]. Between 2–3 months post infection, the female worms start to oviposit around 3000 and 20,000 eggs per day and the eggs remain viable for 2–4 weeks following release into the environment [2].

Traditional diagnostic tools for *T*. *trichiura* infections include stool microscopy including Kato-Katz and FLOTAC, formol-ether concentration and antibody assays [6,7]. Although Polymerase Chain Reaction (PCR) assays have been developed for clinical and public health application, these tests are not feasible for large scale or programmatic surveillance due to high cost, time and skills required for their operation especially in remote and resource limited areas [8]. Moreover, co-infection with multiple parasites make diagnosis challenging in endemic areas [7,9,10]. For *T*. *trichiura*, stool microscopy is used in the diagnosis of uncomplicated cases [9,11], whereas colonoscopy and biopsies are often used for diagnosis of severe cases [10,12]. However, the main challenge of existing diagnostic tools for STH is the fact that they are costly for large scale programmatic surveillance to facilitate timely distribution, scale up or discontinuation of MDA treatments. The availability of novel diagnostic techniques that are rapid and sensitive remain a gap.

The near-infrared spectroscopy (NIRS) technique analyses biological samples through the interaction of light and the chemical profile of those samples. Spectral signatures are characteristic of the chemical profile of samples being analysed and can be used as fingerprints for that sample. NIRS has been previously used to analyse 1) mosquitoes to detect malaria [13], Zika [14], *Wolbachia* [15], Chikungunya [16], 2) Triatomine species to detect *Trypanosoma cruzi* parasites, the causative agent of chagas disease [17] and 3) differentiate *Biomphalaria* species that are intermediate hosts of *Schistosoma mansoni* [18]. NIRS has also been used to age grade and differentiate mosquito species [19,20], differentiate bacteria species [21], detect cancer tumors in patients [22–24], determine blood oxygen saturation and haemoglobin concentration [25,26] and to detect cerebral malaria in children [27]. More recently the technique has been used non-invasively through the skin to detect malaria in human subjects [28].

Experimental mice infected with *T. muris* are a useful model to study *T*. *trichiura* infection in humans to understand the role of the immune system against this infection [5] especially changes related to inflammation, epithelial, host immune responses during in intestinal infection and inflammation [29]. Moreover, *T*. *muris* is morphologically similar and antigenically cross react with *T*. *trichiura* [30,31].

Here, we assessed the capacity of NIRS and machine learning algorithms to detect *T*. *muris* parasites in experimental mice either non-invasively or using faecal and blood samples and validated with serology and parasitology results.

## Methods

### Ethics statement

This study was approved by the James Cook University Animal Ethics Committee (Ethics Approval Number A2677) and the University of Queensland Animal Ethics Committee (AEC Approval Number MED/123/20).

### Experimental infection of mice with *T*. *muris*

To develop and validate *T*. *muris* infection predictive algorithms, two independent experiments were conducted at separate time points. These experiments are hereafter described as experiment 1 and 2. For each experiment conducted, ten mice were orally infected with 30 embryonated *T*. *muris* eggs i.e., the low dose group (L), ten mice were orally infected with 200 embryonated *T*. *muris* eggs i.e. the high dose group (H) and ten mice were orally infected with water i.e. the naïve group (N).

### Collection of faecal and blood samples

Between 1–3 faecal pellets were collected from each mouse at designated time points and stored at 4°C before processing and analysis. Blood was collected from the submandibular vein, and was split into two tubes: one containing a heparin anti-coagulant (whole blood sample) and one empty tube (for serum sample). Serum samples were collected after centrifugation, and either frozen or kept at 4°C until analysis. Faecal, blood and serum samples were collected at 2, 4 and 6 weeks post infection (wpi) during experiment 1 and at 24 hrs, 2 and 6 wpi during experiment 2. In experiment 2, non-invasive scanning using NIRS was conducted at 0 hrs (before infection), 24 hrs, 1, 2, 3, 4, 5 and 6 wpi (Table 1). Timelines in experiment 2 were adjusted to exclude time points assessed in experiment 1 and include time points that were not assessed in experiment 1. This allowed us to develop predictive models that could predict infection on a wider scale.

**Table 1:** Summary of sample and data collection timepoints of experiment 1 and 2.

Experiment 1			Experiment 2		
Faecal	Whole blood	Serum	Faecal	Whole blood	Non-invasive
Week 2	Week 2	Week 2	24hrs	NA	0 hrs_baseline
Week 4	Week 4	Week 4	Week 2	Week 2	24hrs pi
Week 6	Week 6	Week 6	Week 6	Week 6	Week 1, 2, 3, 4, 5,6

#### a. Preparation of faecal, blood and serum samples

Dry faecal samples were first dissolved in 200μL-1000μL of water depending on the size of the pellet and then 2 μL-5 μL of solution was spotted on a glass slide and allowed to air dry for approximately 30 min before analysis with NIRS was conducted. Two μL of whole blood and serum were also spotted on glass slides, and allowed to air dry for 30 minutes-1 hr prior to scanning with NIRS.

### Acquisition of NIRS spectral data from faecal, blood and serum samples

**Fig 1:**
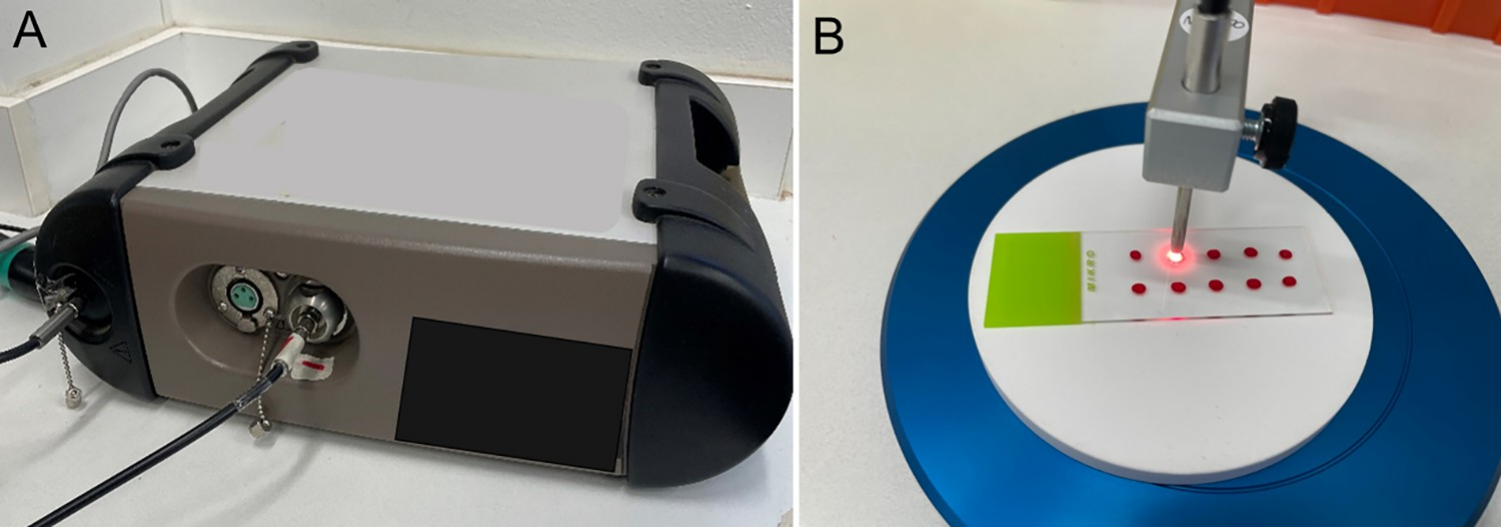
Labspec 4i NIR spectrometer (A) and an example of scanning dry blood on a slide using NIRS (B).

The spectra of faecal, blood and serum were acquired using a Labspec 4i NIR spectrometer (Fig 1A) with an external bifurcated fiber optic probe with 6 illumination fibers with wavelengths ranging from 350-2500nm (ASD Malvern Panalytical, Longmont, CO) as previously described [19]. Briefly, scanning was achieved by directly pointing the fiber optic illuminating probe on to the sample placed 2mm below the light (Fig 1B). At least 10 technical replicates of each biological sample were analysed at each time point.

#### b. Non-invasive NIRS scanning

**Fig 2:**
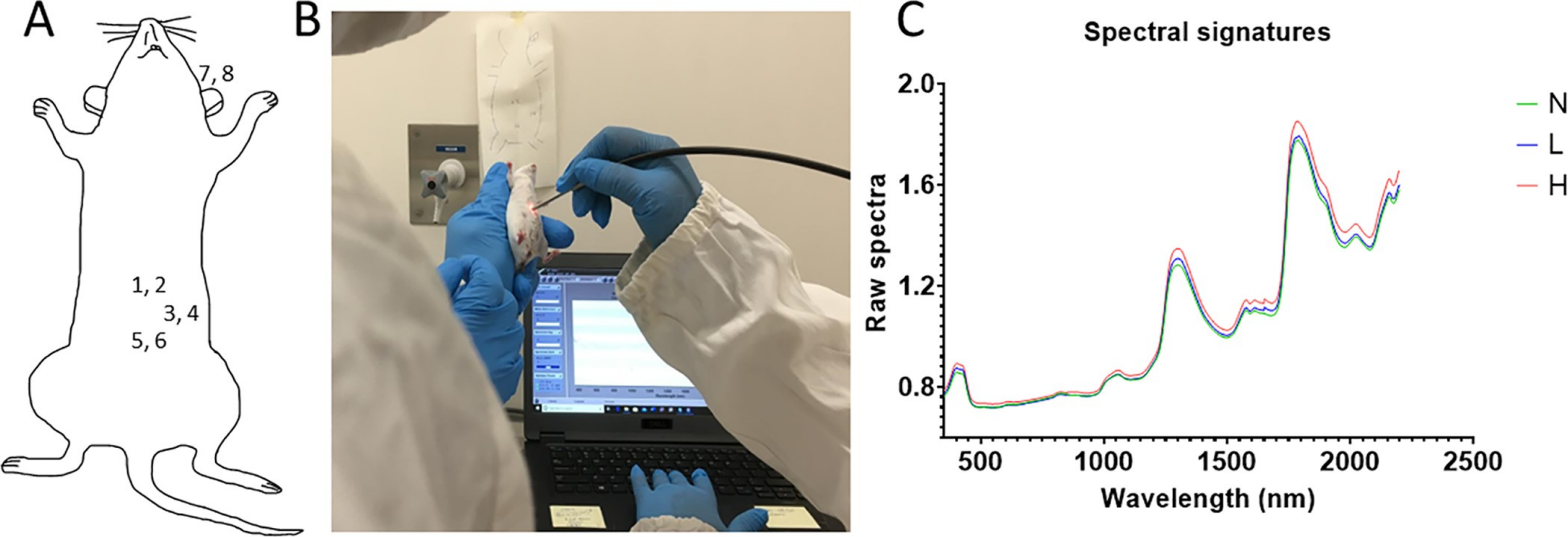
Shows scanning area of the mice; three spots around the large intestine area (1 to 6) and left ear (7, 8) (A). Panel B shows the use of NIRS external fiber optic probe to scan mice and panel C shows the raw average spectral signatures collected non-invasively from all Naïve mice (N), mice from the low dose group (L) and mice from the high dose group (H) of experiment 1.

Infected and uninfected mice were randomly scanned using Labspec 4i NIRS spectrometer by placing the probe so that it was in direct contact with the animal’s body part. The left ear and the area around the large intestine of the mice were scanned (Fig 2A). Adult worms live in the ascending colon and cecum [2] and so the large intestines were scanned to maximize the chance of the NIRS light directly interacting with the parasites in this area. Two replicate spectral signatures were collected from each body part resulting in a total of 8 spectral signatures per mouse per time point scanned. All mice were non-invasively scanned prior to infection and again after 24 hrs, 1, 2, 3, 4, 5 and 6 wpi. Each spectral signature collected was an average of 15 spectral scans. The scanning procedure used for mice and the associated NIRS raw spectra including all body areas scanned are shown in Fig 2A–2C.

### Data analysis

Spectra were converted to.csv format using the ViewSpec Pro version 6.0 software (Analytical Spectral Device Inc., Boulder, CO, USA). Raw data was organized in MS Excel spreadsheet before they were imported for analysis. Data were classified into infection status, sample collection time, body part scanned and mouse number. Data analysis were conducted using JMP Pro 16 software (SAS Institute Inc., Cary, NC, USA, 1989–2021). Several machine learning algorithms were simultaneously fit to initially assess their accuracy on the spectral signatures. These included bootstrap forest, boosted tree, K Nearest Neighbors, artificial neural networks (ANN) and support vector machines. Of these models, ANN was the most suitable for these data. Supervised ANN algorithms were trained to differentiate mice in the N, L and the H groups using faecal, whole blood, serum and non-invasive spectra. A two layer fully connected multi-perceptron ANN was used. All spectral features were directly input into the layers with no feature extraction. We used the TanH function in a fully connected layer of three TanH layers. TanH is a sigmoid activation function that enables the transformation of input variables to a normalised range of values between -1 and 1. This allows the computation/input of variables on different scales in one ANN. A random seed was set to allow reproducibility of the ANN results. ANN are stochastic in nature and thus a repeat of the analysis with the same data, features and software yields slightly different results but not significantly different. For reproducibility and to ensure all the methods are captured correctly, a seed is used to guarantee the exact results are reproduced. ANN models were trained using the Random Holdback, and a single TanH layer with three nodes boosted 20 times. Spectra signatures from 500–2350 nm were used as predictors in the model, whereas infection status was used as the response factor.

ANN models for predicting infection in faecal and whole blood were trained using samples from one experiment and resultant predictive algorithms were validated 3 times. The first validation was done using spectra of mice from the same experiment that were seen by the model but not used in the model (Validation 1). The second validation was done on spectra from mice that were from the same experiment but were not seen by the model (Validation 2) and the 3rd validation comprised of spectra of all the 30 mice from the second experiment (Test set). Serum and non-invasive scanning were only collected in one experiment but data training, validation and test set was grouped based on biological replicates at various time points shown in Table 1 above. The spectra of serum from experiment 1 were split into training set (60% with 258 signatures from 6 mice across 3 time points), validation set (20% with 84 signatures from 2 mice across 3 time points) and test set (20% with 90 signatures from 2 mice across 3 time points). Similarly, all spectra collected non-invasively during experiment 2 were divided into training set (60% with 1152 signatures from 6 mice across 8 time points), validation set (20% with 384 signatures from 2 mice across 8 time points) and test set (20% with 384 signatures from 2 mice across 8 time points).

Further statistical analyses were conducted in GraphPad Prism version 9.3.1 (GraphPad Software Inc., CA). The average of spectral signatures collected from each mouse from each group were tested for normality using a Shapiro Wilk test. One-way ANOVA or Kruskal-Wallis test was conducted to examine any significant differences between doses. A post hoc Tukey Honest Significant Differences (Tukey HSD) or Dunn’s multiple comparison test was used to assess whether there were significant differences between the three dose groups. Statistical differences between the three groups were first assessed regardless of the period the mice had been infected and thereafter for each week the mouse was infected and visualized using box plots.

### Analysis of worm burden and serology

Where applicable, the number of worms were enumerated at necropsy as previously described [32]. *Trichuris*-specific serum IgG1 and IgG2a titres were measured by enzyme-linked immunosorbent assays (ELISA) on plates coated with *T*. *muris* antigen (5μg/mL) as previously described [33]. The same statistical analysis and visualization described above were used for serology using titres of serum.

## Results

### Worm burden

At necropsy of experiment 1; all the mice from the naïve group had no worms, from the low dose group; 4 mice had no worms, 5 mice had worms ranging from 1–21, and all but 2 mice from the high dose group had completely cleared their worms. On the other hand, at necropsy of experiment 2 all the mice had completely cleared their worms.

### ELISA

**Fig 3:**
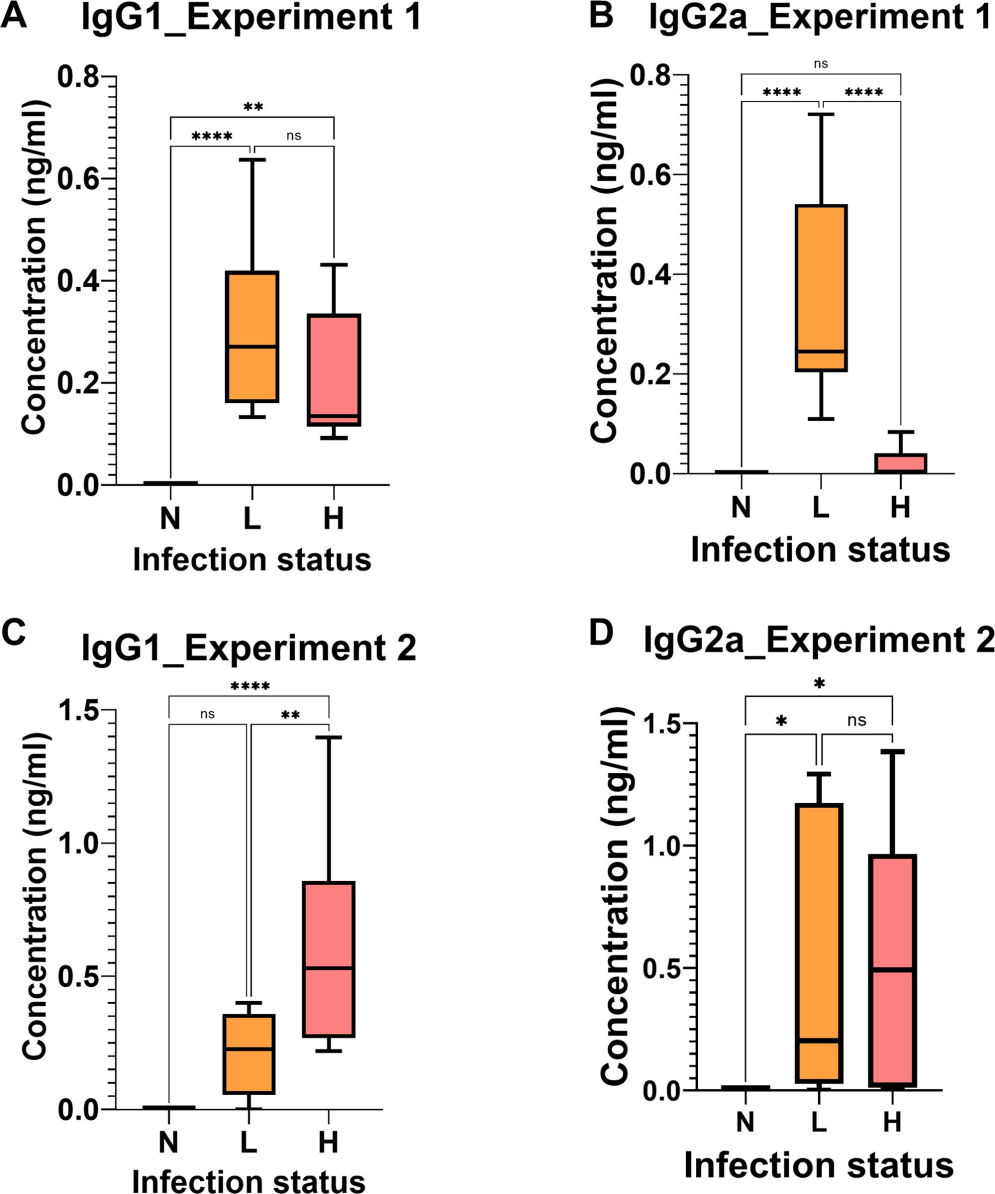
Experiment 1 IgG1 (A) and experiment 1 IgG2a (B) concentrations of all mice from the L and H groups except mouse 5 in the L dose and mouse 1 in the H dose, experiment 2 IgG1 (C) and experiment 2 IgG2a (D) concentrations of all mice from the L and H dose groups.

At the end of experiment 1, *Trichuris*-specific serum IgG1 titres (1:640) for the L and H group were on average 0.304 and 0.212 absorbance units, respectively (Fig 3A). Whereas *Trichuris*-specific serum IgG2a titres (1:40) for L and H groups was on an average 0.363 and 0.0194 absorbance units, respectively (Fig 3B). A significant difference was observed for the L and H groups for IgG2a (P<0.0001) but not for IgG1 (P< 0.24).

Similarly, for experiment 2 *Trichuris*-specific serum IgG1 titres (1:320) for the L and H groups was 0.266 and 0.62 absorbance units, respectively (Fig 3C) and these titres were significantly different (P<0.001). The IgG2a titres (1:20) for the L and the H group was 0.47 and 0.5 absorbance units, respectively (Fig 3D) and the difference was not significant (P<0.98).

### NIR differentiation of infected from uninfected mice

#### Faecal

**Fig 4:**
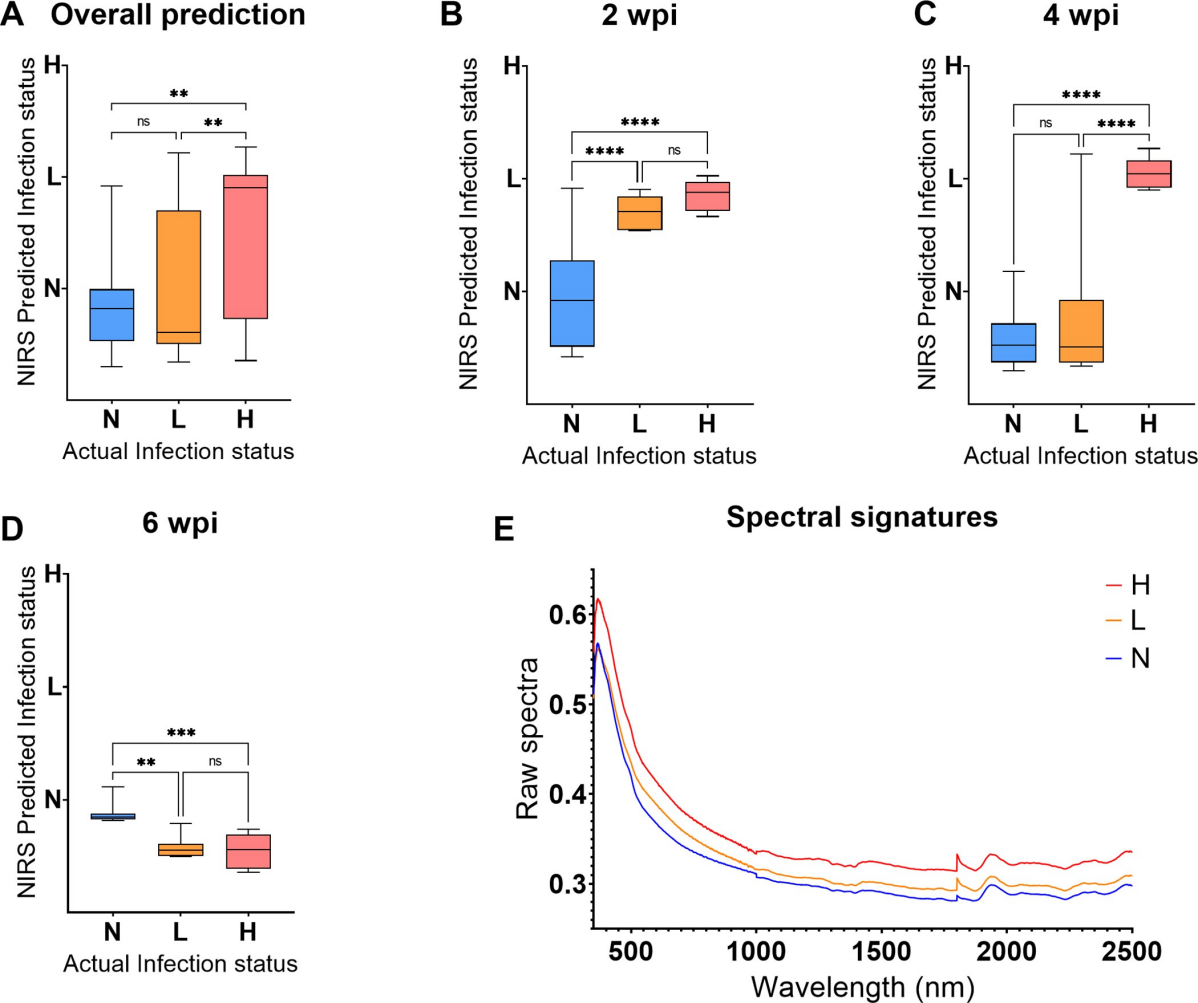
Differentiating N, L, H mice groups using spectra collected from faecal samples of the mice that were used to validate the training model (test set). Results are shown for overall differentiation regardless of the period of infection (A), 2, 4 and 6 wpi (B-D, respectively) and raw spectra of the infected and uninfected mice collected at 2 wpi (E). Statistical significance between the three groups is shown with an asterisk.

An algorithm trained from spectra of faecal samples collected from mice in experiment 2 was used to predict *T*. *muris* infection status of faecal samples collected from mice in experiment 1. The model predicted infection in mice from experiment 1 regardless of the period the mice were infected for. Overall, mice from the N group were significantly different from those from the H group (P<0.001, N = 26) and mice from the L group were significantly different from those from the H group (P<0.002, N = 28). However, the L and N group did not significantly differ (P>0.99) (Fig 4A). At 2 wpi, mice from the N group could be differentiated from mice from both the L (P<0.0001), and H group (P<0.0001), however the two infected groups could not be differentiated from each other (P = 0.55) (Fig 4B). At 4 wpi, mice from the N group could be differentiated from those in the H group (P<0.0001) but not the L group (P = 0.58) (Fig 4C). Consistent with the ELISA results, at 6 wpi, the L (P<0.001) and H (P<0.0008) groups were significantly different from the N group but the two groups could not be differentiated (P>0.99) (Fig 4D). The NIRS raw spectral signatures used to develop a model for differentiating uninfected from infected mice using faecal samples collected at 2 wpi are shown in Fig 4E. Absorption differences from the raw spectra collected from Trichuris infected and uninfected mice (Fig 4E) were seen, regardless of the intensity of the infection.

#### Whole blood

**Fig 5:**
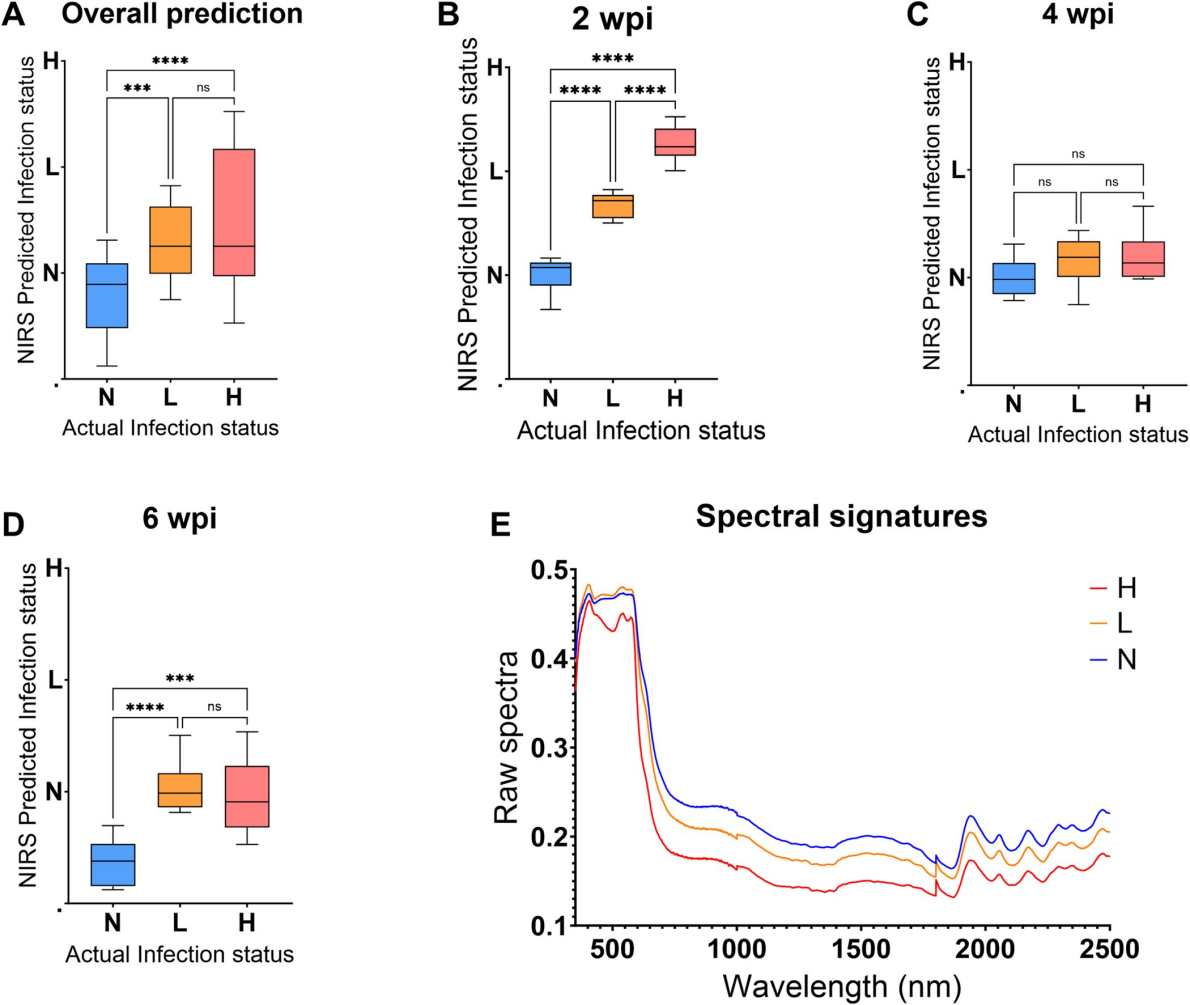
Differentiating N, L, H mice groups using spectra collected from whole blood samples of the test set. Results are shown for overall differentiation regardless of the period of infection (A), 2, 4 and 6 wpi (B-D, respectively) and the raw spectra of the infected and uninfected mice collected at 2 wpi (E). Statistical significance between the three groups is shown with an asterisk.

Machine learning algorithm trained using spectra of blood samples collected from mice in experiment 2 were used to predict *T*. *muris* infection status of the blood samples collected from mice in experiment 1. The model predicted infection regardless of the period the mice were infected for. Overall, mice from the N group were significantly different from those from the H group (P<0.0001, N = 28) and L group (P<0.0003, N = 29). However, the L and H group did not significantly differ (P>0.99, Fig 5A). At 2 wpi mice from the N, L and H groups could be differentiated from each other (P<0.0001) (Fig 5B) but at 4 wpi, mice from the three groups could not be differentiated from each other (Fig 5C). At 6 wpi, mice from the N group were significantly different from those from the H group (P<0.0002) and L group (P<0.0001) (Fig 5D). However, the L and H group did not significantly differ (P = 0.81) (Fig 5D). The NIRS raw spectral signatures used to develop a model for differentiating uninfected from infected mice using blood samples collected at 2 wpi are shown in Fig 5E. Absorption differences from the raw spectra collected from Trichuris infected and uninfected mice were seen, regardless of the intensity of the infection (Fig 5E).

#### Serum

**Fig 6:**
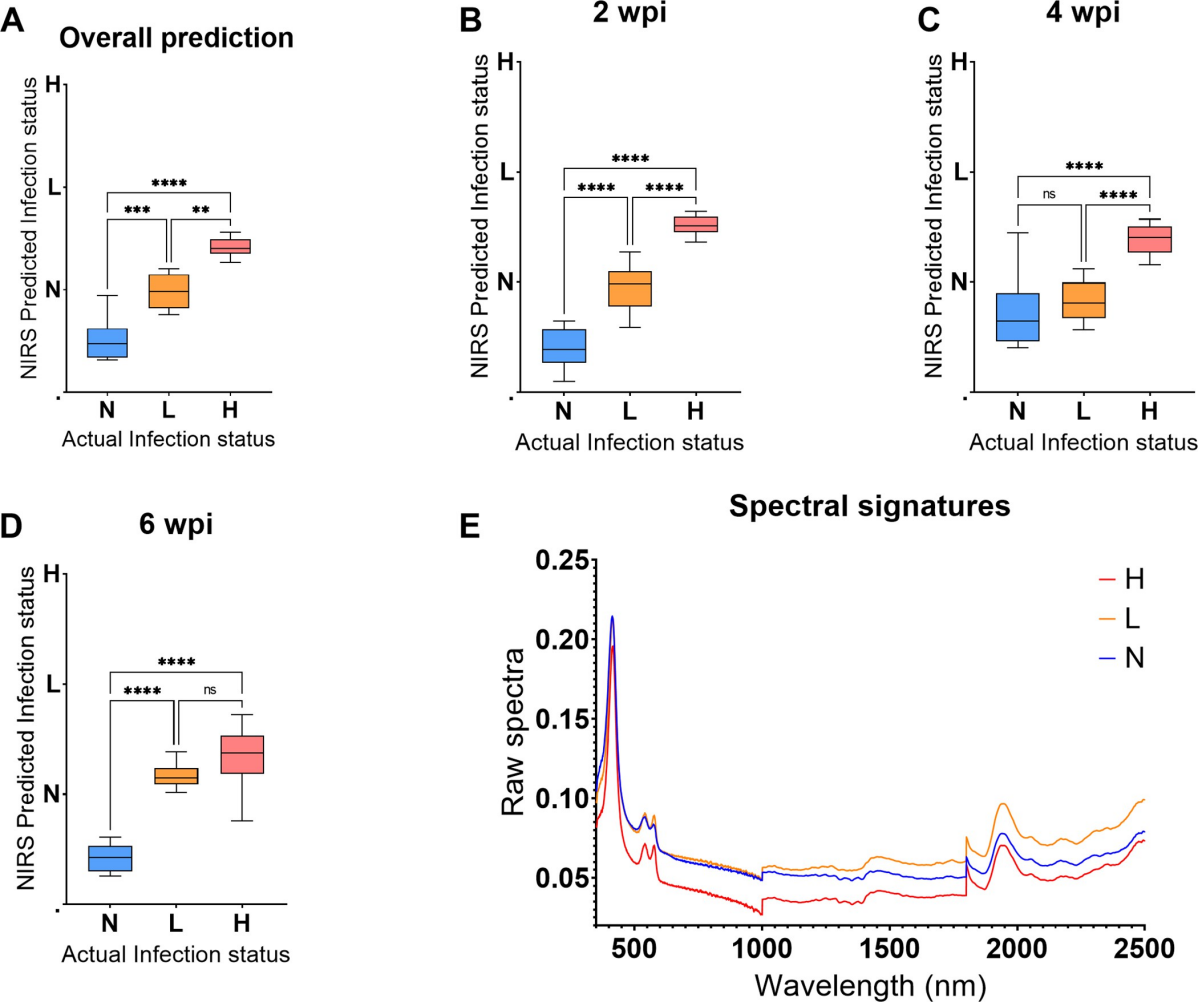
Differentiating N, L, H mice groups using spectra of serum samples from the test set regardless of the infection period. Results are shown for overall differentiation regardless of the period of infection (A), 2, 4 and 6 wpi (B-D, respectively) and the raw spectra of the infected and uninfected mice collected at 2 wpi (E). Statistical significance between the three groups is shown with an asterisk.

A training model using spectra of serum samples collected from mice in experiment 1 was used to predict *T*. *muris* infection status of samples that were excluded from the training model. The model predicted infection in mice regardless of the period the mice were infected for. The model differentiated mice from the N, L and H groups from each other regardless of the period post infection (P<0.0001, Fig 6A) and also at 2 wpi (P<0.0001, Fig 6B). However, at 4 wpi, mice from the N group could be differentiated from those in the H group only (P<0.0001) but not the L group (P = 0.46) (Fig 6C). At 6 wpi, the L (P<0.0001) and H (P<0.0001) groups were significantly different from the N group but the two groups could not be differentiated (P = 0.15) (Fig 6D). The NIRS raw spectral signatures used to develop a model for differentiating uninfected from infected mice using serum collected at 2 wpi are shown in Fig 6E. Absorption differences from the raw spectra collected from Trichuris infected and uninfected mice were seen, regardless of the intensity of the infection (Fig 6E).

#### Non-invasive scanning

**Fig 7:**
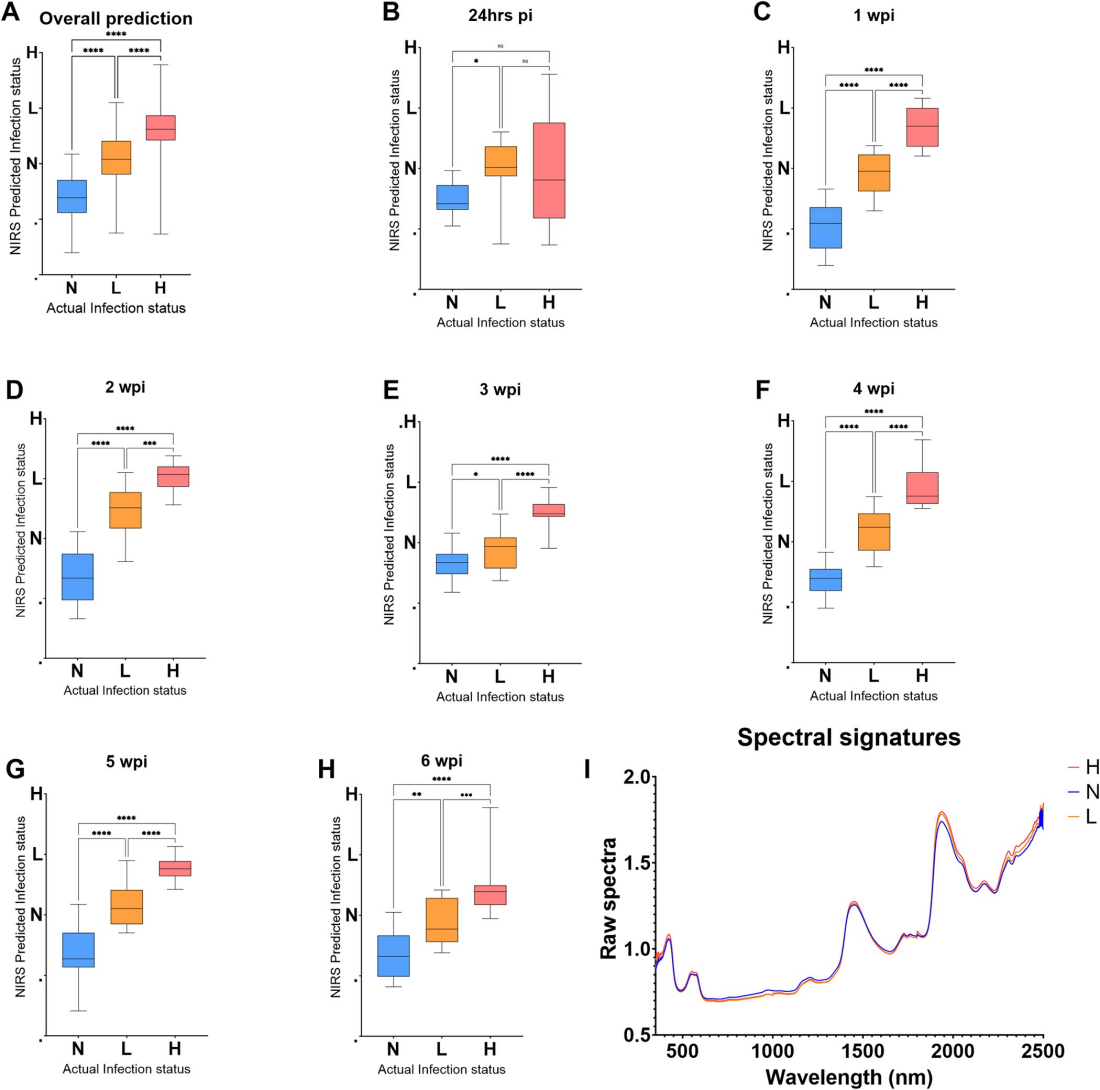
Differentiating N, L, H mice groups using spectra collected non-invasively from the test set regardless of period of infection. Results are shown for overall differentiation regardless of the period of infection (A), 24 hrs pi, 1, 2, 3, 4, 5 and 6 wpi (B-H, respectively) and raw spectra of the infected and uninfected mice collected at 2 wpi (I). Statistical significance between the three groups is shown with an asterisk.

A model trained using spectra collected non-invasively through the skin of mice from experiment 2 was used to predict the *T*. *muris* infection status for mice that were excluded from the model at 8 different time points. The trained model predicted infection in mice regardless of the period the mice were infected for. Moreover, mice from N, L and H groups were differentiated from each other (P<0.0001, Fig 7A) regardless of the body part that was scanned (cecum or ear). At 24 hrs post infection, mice from the N group were significantly different from mice from the L group (P<0.03) but not the H group (P = 0.07), and mice from the two infected groups could not be differentiated (P = 0.95) (Fig 7B). However, from 1–6 wpi, mice from the three groups could be differentiated from each other (P<0.0001) (Fig 7C–7H). Spectral signatures collected at 2 wpi are shown in Fig 7I. Absorption differences from the raw spectra collected from Trichuris infected and uninfected mice were seen relative to the intensity of the infection (Fig 7I).

### Comparative cost and time assessment

Scanning samples using NIRS takes approximately 5–10 seconds depending on the sample being scanned. No reagents are required, and only minimal sample processing procedures are involved. Following development of predictive algorithms, results are obtained in real time. Based on this study, we estimated that, an average of 3750 samples can be scanned in 1 day by a single person. Although current outlay costs for the NIR instrument used in this analysis is approximately USD55000, these costs can be reduced to <USD1 after analysing approximately 30,000 samples. Alternatively, NIR instrumentation costs could be reduced through utilization of smaller handheld units whose average cost is USD2000/unit. However, these units are yet to be assessed. Comparatively, analysis using the Kato Katz technique takes approximately 23–34 mins/sample with an average cost of $1.89/sample [34], the FLOTAC technique takes 15–26 mins to run and each sample costs $2.6 to analyse [34] and PCR takes approximately 3.5 hrs/assay with the cost of US$6.43/assay [35]. Table 2 summarises the costs, time and accuracy of NIRS relative to that of Kato Katz, FLOTAC and PCR techniques.

**Table 2:** Cost and time analysis of NIRS versus existing Trichuris diagnostic techniques.

Feature	NIRS (Faecal, blood and serum)	NIRS Non-invasive	Kato Katz	FLOTAC	PCR
Time/sample analysed	10 sec	5 sec	23-34min [11,34]	15-26min [11,34]	Approximately 3.5hrs/assay (STH) [36]
Cost of materials/samples	0	0	US$ 1.73–2.06 [11,34]	US$ 2.35–2.83 [11,34]	US$6.43/assay (STH) [36]
Sensitivity	100% at 2 wpi (faecal and blood) 95% at 2 wpi (serum)	97% at 2 wpi	67–91% (Trichuris) [37] 70–96% (Ascaris) [37] 12–95% (Hookworms) [37]	67–91% (Trichuris) [37] 61–63% (Ascaris) [37] 83–88% (Hookworms) [37]	93.1–96.1% (Trichuris) [35] 87.4–92.4% (Ascaris) [35] 89.8–93.8% (Hookworms) [35]
No. of samples analyzed/day/person	2500	5000	26–46	16–24	-

## Discussion

This study assessed the diagnostic performance of NIR benchtop spectrometer to differentiate *T*. *muris* infected from uninfected mice using faecal, blood and serum samples. The study also provided an opportunity for us to test the potential of the technique to non-invasively detect *T*. *muris*, through the skin of experimental mice.

We hypothesized that whipworm infection alters the immune responses of mice characterized by increased epithelial cell turnover, mucin secretion, immune responses, inflammation and host defense mechanisms in intestinal infection [5,29]. These changes together with the presence of the worms and eggs are expected to generate a spectral signature with unique absorption bands for the infected and uninfected mice [13,38]. Our findings indicate that NIRS coupled with machine learning algorithms can differentiate *T*. *muris* infected from uninfected mice using faecal, blood or by simply scanning the skin of the mice. This result was consistent with the ELISA results shown in Fig 3 where *Trichuris*-specific serum levels correlated with NIR infection predictions.

Our results indicate that NIRS can differentiate *T*. *muris* infected and uninfected mice as early as 2 wpi. This finding is likely due to the fact that larvae of whipworms in the cecum and proximal colon wall dwell in the epithelial layer and undergo two moults between 9–11 days (L2) and 17 days (L3) post infection [39]. It has also been reported that mice resistant to *T*. *muris* expel their worms between 12- and 21-days post infection [40] which could explain the ability of NIRS to differentiate infected from uninfected groups 2 wpi using fecal samples. This result is impressive as it means the tool could potentially be used to detect these parasites early prior to onset of symptoms.

Faecal, blood and serum samples could not be differentiated into the L group and the H group at 6 wpi. This result was consistent with the ELISA results shown in Fig 3A where IgG1 levels were not significantly different between the L and H groups at this time point. Previous studies have shown that the initial antigen dose can have an effect on the outcome of infection with *T*. *muris* and that low level of infection can have a significant effect on the polarization of the CD4 response by producing high amount of Th2 cytokine [41]. Moreover, the high number of worms observed in the cecum can trigger a chronic infection in the L group making them look similar to the mice in the H dose group.

We also demonstrated that spectral signatures collected non-invasively through the skin of mice could be used to detect the presence of *T*. *muris* infection in experimental mice. The spectral signatures collected at 1, 2, 3, 4, 5 and 6 wpi differentiated mice in the three groups providing evidence of the potential application of the technique not only for detecting early onset of infection but also as a justifiable platform for scanning asymptomatic patients. Detecting infection early can prompt timely treatment and guide MDA strategies appropriately, especially, where entire risk groups are offered preventive treatment. Non-invasive scanning does not require sample processing procedures nor reagents to operate. Following the purchase of NIR instrument, current costs required to produce a test for STH could reduce to <USD1. We have also shown that *T*. *muris* infection can be detected by scanning either the cecum or the ear of the mouse. The fact that NIRS could detect infection in ears confirmed our hypothesis that the technique is likely detecting immune responses as well as the presence of the parasite to differentiate infected from uninfected groups. Two recent studies by our group indicated that NIRS can detect malaria in experimental mice and human subjects through their ears [28]. Moreover, previous studies on disease vectors showed that NIRS can detect infection such as Malaria [13], Zika [14], Wolbachia [15] and Chikungunya [16] by simply scanning heads/thoraces and abdomens of mosquitoes or *T*. *cruzi* by scanning midguts, rectum or excreta of Triatomine species [17].

Although it is unclear which chemical changes were responsible for the differentiation, we present evidence using faecal, blood and non-invasive scans that NIRS could potentially be used as an early surveillance tool for intestinal worms. Current *T*. *trichiura* diagnostic methods are expensive, technically demanding, time-consuming, require specialized expertise and have a limited capacity to be used for large scale surveillance in endemic or areas aiming for elimination. For example, both FLOTAC and Kato Katz techniques are the current mainstay diagnostic tools for STH. These tools can be costly and time consuming for large scale surveillance programs [11,34,42–44]. Antibody assays and formol-ether concentration tests are time consuming, use hazardous chemicals and are technically demanding [37] whereas PCR assays are costly, time-consuming, technically demanding, and require specialized expertise [8]. Due to the time required to process large samples, lack of sensitivity and associated costs, alternative surveillance tools are currently being sought that can guide scale up/down of MDA programs. NIRS is environmentally friendly, it does not require reagents and only minimal sample processing procedures are required. NIRS takes only 5–10 sec to scan a sample allowing thousands of samples to be assessed in a day by 1 person with minimal training.

Our laboratory results provide evidence that NIRS has potential to be used for early detection and surveillance of STH infections particularly for large scale surveillance studies. Future community-based studies are recommended to demonstrate the potential of NIRS to detect STH under real world settings. These studies will facilitate the development of robust predictive models and provide insights into the potential of the technique to detect STH co-infections and malaria in communities where mixed infections occur. Nevertheless this proof-of-concept study has provided insights into the role NIRS and machine learning algorithms could play in the detection of STH infections.

## Supporting information

S1 Text **Fig A.** Differentiating N, L, H mice groups using the spectra collected from faecal samples of the mice that were used for training the model regardless of the period of infection, at 24 hrs pi, 2 and 6 wpi. Related to Fig 4. **Fig B.** Differentiating N, L, H mice groups using the spectra collected from whole blood samples of the mice that were used for training the model at 2 and 6 wpi. Related to Fig 5. **Fig C.** Differentiating N, L, H mice groups using the spectra collected from serum samples of the mice that were used for training the model at 2, 4 and 6 wpi. Related to Fig 6. **Fig D.** Differentiating N, L, H mice groups using the spectra collected non-invasively for the mice that were used for training the model at 24 hrs pi and at 1–6 wpi. Related to Fig 7. **Table A.** Results of serology (Enzyme-linked immunosorbent assays (ELISA) and the number of worms at necropsy for the first study (experiment 1) and the second study (experiment 2) for the mice from the naïve group (N), low dose group (L) and the high dose group (H). Related to Fig 3.
(DOCX)
