# Supplementary material for: Near-infrared spectroscopy and machine learning algorithms for rapid and non-invasive detection of Trichuris
Source: PLoS Negl Trop Dis. 2023 Nov 13;17(11):e0011695. doi: 10.1371/journal.pntd.0011695 (PMC10681298; doi:10.1371/journal.pntd.0011695)
Supplement: S1 Text — Fig A. Differentiating N, L, H mice groups using the spectra collected from faecal samples of the mice that were used for training the model regardless of the period of infection, at 24 hrs pi, 2 and 6 wpi. Related to Fig 4. Fig B. Differentiating N, L, H mice groups using the spectra collected from whole blood samples of the mice that were used for training the model at 2 and 6 wpi. Related to Fig 5. Fig C. Differentiating N, L, H mice groups using the spectra collected from serum samples of the mice that were used for training the model at 2, 4 and 6 wpi. Related to Fig 6. Fig D. Differentiating N, L, H mice groups using the spectra collected non-invasively for the mice that were used for training the model at 24 hrs pi and at 1–6 wpi. Related to Fig 7. Table A. Results of serology (Enzyme-linked immunosorbent assays (ELISA) and the number of worms at necropsy for the first study (experiment 1) and the second study (experiment 2) for the mice from the naïve group (N), low dose group (L) and the high dose group (H). Related to Fig 3. (DOCX) [file pntd.0011695.s001.docx]

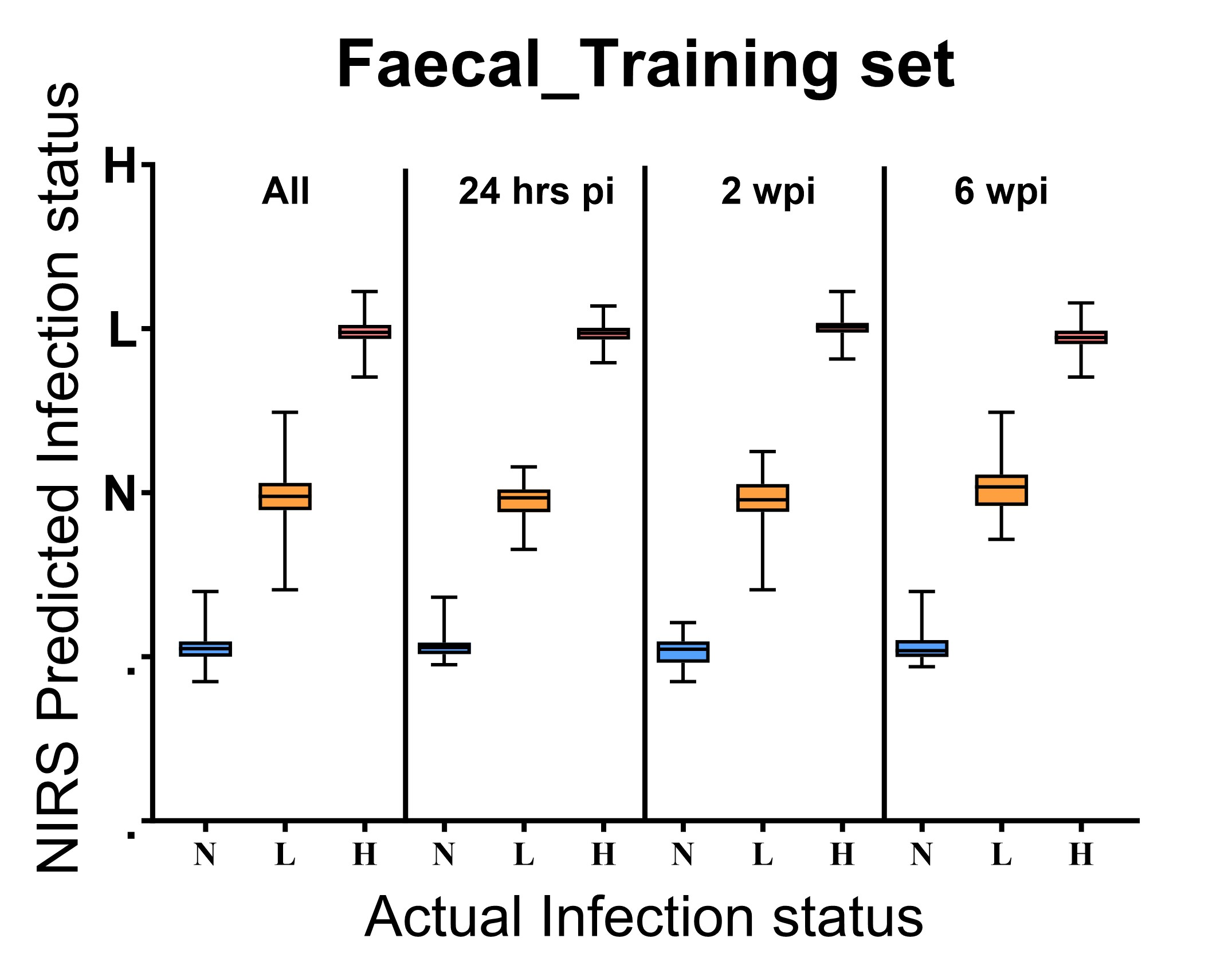


Fig A. Differentiating N, L, H mice groups using the spectra collected from faecal samples of the mice that were used for training the model, regardless of the period of infection, at 24 hrs pi, 2 and 6 wpi. Related to Fig 4.


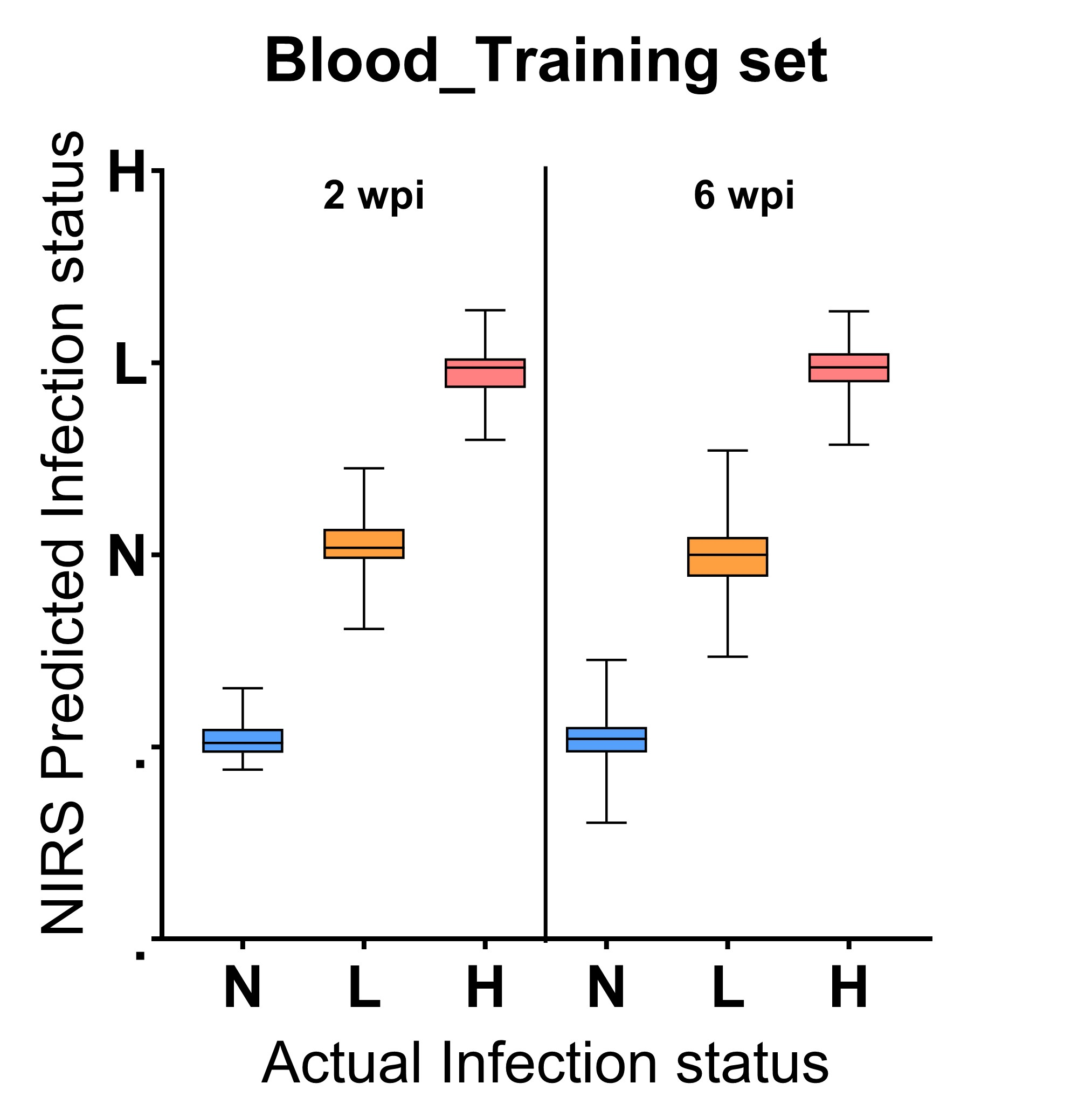


Fig B. Differentiating N, L, H mice groups using the spectra collected from whole blood samples of the mice that were used for training the model at 2 and 6 wpi. Related to Fig 5.


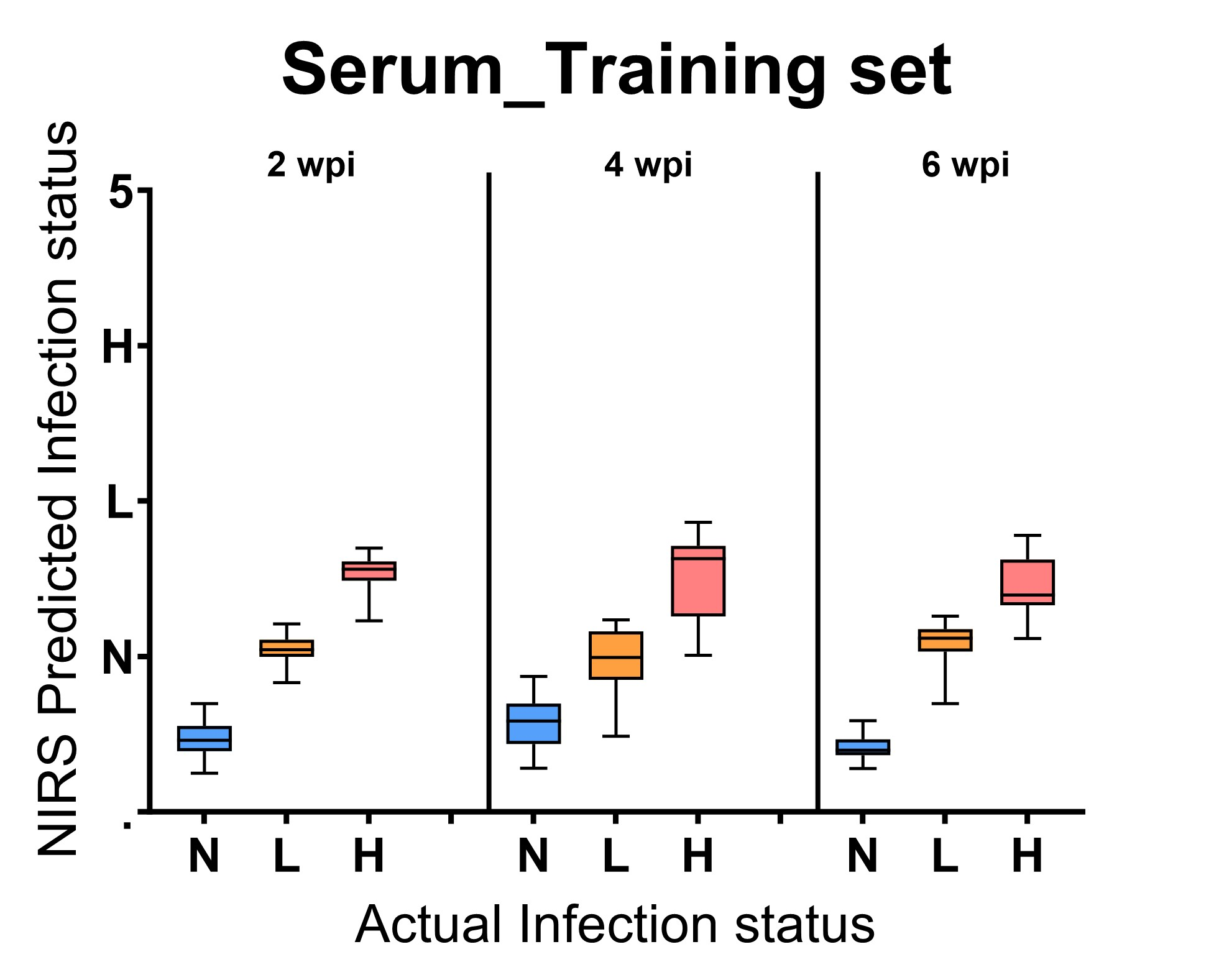


Fig C. Differentiating N, L, H mice groups using the spectra collected from serum samples of the mice that were used for training the model at 2, 4 and 6 wpi. Related to Fig 6.


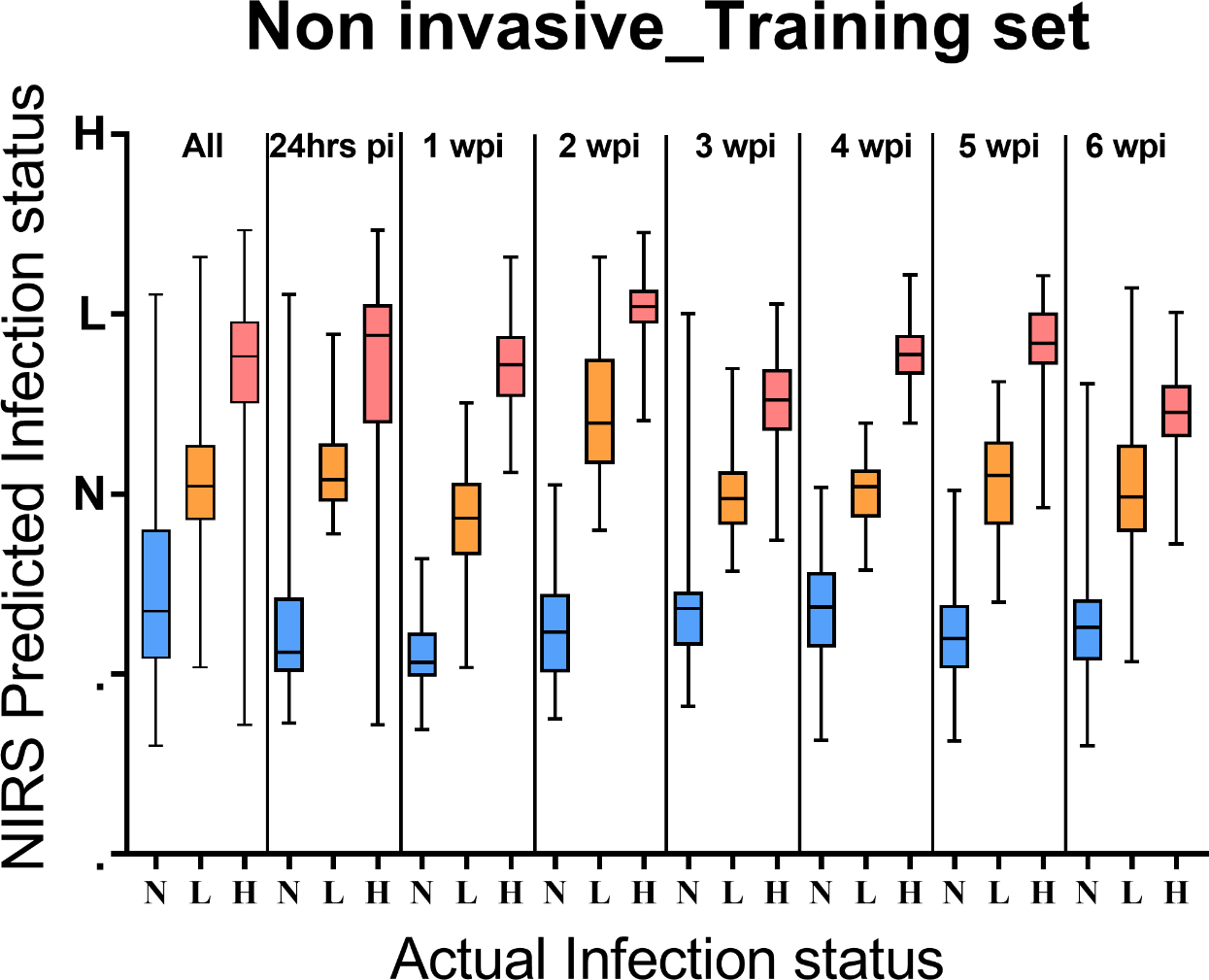


Fig D. Differentiating N, L, H mice groups using the spectra collected non-invasively for the mice that were used for training the model at 24 hrs pi and at 1-6 wpi. Related to Fig 7.

Table A. Results of serology (Enzyme-linked immunosorbent assays (ELISA)) and the number of worms at necropsy for the first study (experiment 1) and the second study (experiment 2) for the mice from the naïve group (N), low dose group (L) and the high dose group (H). Related to Fig 3.

| **Experiment**  **1** | **IgG1**  **(1:640)** | **IgG2a**  **(1:40)** | **Number**  **of worms** |  | **Experiment**  **2** | **IgG1**  **(1:320)** | **IgG2a**  **(1:20)** | **Number**  **of worms** |
| --- | --- | --- | --- | --- | --- | --- | --- | --- |
| L1 | 0.431 | 0.245 | 0 |  | L1 | 0.347 | 0.142 | 0 |
| L2 | 0.148 | 0.22 | 0 |  | L2 | 0.049 | 1.292 | 0 |
| L3 | 0.271 | 0.187 | 0 |  | L3 | 0.312 | 0.092 | 0 |
| L4 | 0.408 | 0.11 | 3 |  | L4 | 0.4 | 0.005 | 0 |
| L6 | 0.637 | 0.548 | 7 |  | L5 | 0.211 | 1.163 | 0 |
| L7 | 0.133 | 0.721 | 21 |  | L6 | 0.241 | 0 | 0 |
| L8 | 0.288 | 0.481 | 1 |  | L7 | 0.057 | 1.209 | 0 |
| L9 | 0.172 | 0.534 | 0 |  | L8 | 0 | 0.034 | 0 |
| L10 | 0.252 | 0.221 | 6 |  | L9 | 0.058 | 0.266 | 0 |
|  |  |  |  |  | L10 | 0.391 | 0.503 | 0 |
|  |  |  |  |  |  |  |  |  |
| H2 | 0.135 | 0.054 | 0 |  | H1 | 0.236 | 0.569 | 0 |
| H3 | 0.107 | 0.004 | 0 |  | H2 | 0.554 | 0.049 | 0 |
| H4 | 0.321 | 0.084 | 0 |  | H3 | 0.858 | 1.384 | 0 |
| H5 | 0.431 | 0.005 | 0 |  | H4 | 0.86 | 0.416 | 0 |
| H6 | 0.129 | 0 | 2 |  | H5 | 0.219 | 0.942 | 0 |
| H7 | 0.092 | 0 | 0 |  | H6 | 1.396 | 0.008 | 0 |
| H8 | 0.221 | 0 | 0 |  | H7 | 0.279 | 0.592 | 0 |
| H9 | 0.122 | 0 | 2 |  | H8 | 0.838 | 0.007 | 0 |
| H10 | 0.35 | 0.028 | 0 |  | H9 | 0.506 | 0.013 | 0 |
|  |  |  |  |  | H10 | 0.454 | 1.038 | 0 |
| naïve | 0 | 0 | 0 |  |  |  |  |  |
|  | | | | | naïve | 0 | 0 | 0 |
